# Cortical contributions to the auditory frequency-following response revealed by MEG

**DOI:** 10.1038/ncomms11070

**Published:** 2016-03-24

**Authors:** Emily B. J. Coffey, Sibylle C. Herholz, Alexander M. P. Chepesiuk, Sylvain Baillet, Robert J. Zatorre

**Affiliations:** 1 Montreal Neurological Institute, McGill University, Montreal, Québec, Canada H3A 2B4; 2 Centre for Research on Brain, Language and Music (CRBLM), Montreal, Québec, Canada H3G 2A8; 3 International Laboratory for Brain, Music, and Sound Research (BRAMS), Montreal, Québec, Canada; 4 German Center for Neurodegenerative Diseases (DZNE), Bonn 53175, Germany

## Abstract

The auditory frequency-following response (FFR) to complex periodic sounds is used to study the subcortical auditory system, and has been proposed as a biomarker for disorders that feature abnormal sound processing. Despite its value in fundamental and clinical research, the neural origins of the FFR are unclear. Using magnetoencephalography, we observe a strong, right-asymmetric contribution to the FFR from the human auditory cortex at the fundamental frequency of the stimulus, in addition to signal from cochlear nucleus, inferior colliculus and medial geniculate. This finding is highly relevant for our understanding of plasticity and pathology in the auditory system, as well as higher-level cognition such as speech and music processing. It suggests that previous interpretations of the FFR may need re-examination using methods that allow for source separation.

## Abstract (web summary)

Auditory brainstem response (ABR) is used to study temporal encoding of auditory information in music and language. This study utilizes magnetoencephalography to localize both cortical and subcortical origins of the sustained frequency following response (FFR), the ABR component that encodes the periodicity of sound.

## Graphical abstract

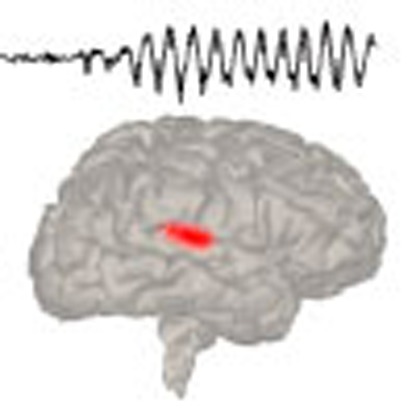

Auditory brainstem responses (ABRs) are time-locked neural reactions to sound that are recorded from the scalp using electroencephalography (EEG)^1^. Because they are uniquely suited to studying fine temporal encoding in the intact human auditory system, an accumulation of ABR-based results now supports some of our general understanding of the auditory system, particularly as it relates to the human pursuits of language and music.

ABRs to complex sounds such as speech and musical tones have two main characteristics: a transient response to a sudden sound onset or a click, and a sustained frequency-following response (FFR) to periodicity^1^. The click or onset response is a relatively straightforward progression of auditory information from the auditory nerve to the cortex and is valuable particularly for clinical diagnosis. Its neural origins are quite well understood^2^,^3^.

The FFR portion of the ABR is of a different nature to the onset response, as it appears to provide information to different higher-level systems and co-vary with distinct behavioural and clinical measures^1^,^4^. As such, it is thought to reflect the basic neural representation of periodic sound on which higher-level processing of language and music is based^5^. As the name ‘auditory brainstem response' implies, the ABR (including the FFR) is generally thought to arise from the summation of signals from interconnected subcortical sources in auditory brainstem nuclei^6^. However, measures of the FFR's strength, consistency and frequency content are susceptible to modulation by processes with known cortical involvement, including learning^7^, task-related suppression^8^ and selective attention^9^; FFR measures also relate more strongly to complex cognitive skills such as reading than to basic sound perception^10^. These results have been interpreted as local effects within the brainstem or as reflecting efferent modulation of subcortical processes from the cortex; however, the neural origins of the FFR are very much an open question^6^,^11^.

A large number of recent studies in basic and applied research areas have taken FFR measures as proxies for measuring subcortical activity. For example, the locus, extent and variety of experience-based plasticity in the auditory system have been inferred partially from FFR studies^12^. These include results from studies demonstrating that the FFRs of musicians and tonal language speakers are more robust than those of non-musicians, and that sound representation is malleable to short-term training^13^,^14^,^15^,^16^,^17^,^18^. FFR studies have also provided insights into auditory processing changes during early development and in ageing^19^,^20^,^21^,^22^,^23^. Music training in adolescence confers benefits for language skills, and this is thought to occur in part due to its effect on subcortical sound processing^24^. Performance deficits in speech and language processing in older adulthood are attributed to a loss of temporal precision of sound processing at the brainstem level^25^,^26^. Understanding speech in noisy conditions has also been studied using FFRs. Degradation of the FFR in the presence of noise is reduced following perceptual training^26^,^27^, suggesting that training causes changes to the neural encoding of complex sounds by improving neural synchrony in the auditory brainstem. Various studies have also reported differences in the FFR of clinical populations, such as children with learning disabilities and autism, and attributed them to functional impairments at the brainstem level^28^,^29^,^30^. FFR measures have also proven sufficiently sensitive for use as biomarkers to identify and evaluate the treatment of disorders that feature abnormal sound processing^31^,^32^,^33^,^34^,^35^. Measures such as the strength and consistency of sound representation in the FFR or of transient peaks after the ABR onset response are used in all of these studies.

Magnetoencephalography (MEG) is suitable for studying the anatomical origins of FFRs, as neural signals remain largely unaffected by the geometry and electromagnetic properties of the head tissues through which they pass, making source localization from MEG data more accurate than EEG data^36^. Attempts to use MEG to study ABRs date back over two decades^37^,^38^,^39^, but robust and reproducible responses to clicks were only recently reported^40^. A previous study^40^ showed the onset response ascending the auditory system by using equivalent current dipoles, a technique which they note is unsatisfactory for later ABR waves with simultaneously active generators; hence, the origins of the FFR remain unknown. Methods for MEG analysis have evolved rapidly. In particular, distributed source modelling analysis offers the possibility of localizing and separating simultaneous sources—a prerequisite for looking at the FFR. It is now possible for MEG to accurately localize deeper structures such as the hippocampus, amygdala and thalamus (for example, Attal and Schwartz^41^; Dumas *et al*.^42^), suggesting that with sufficient signal to noise, brainstem activity might also be measured.

Clarifying the neural origins of the FFR is therefore of broad interest in auditory cognitive neuroscience. Also, our understanding of early and rapidly time-varying processes in the auditory system is being shaped by the widespread assumption of an exclusive subcortical FFR origin. Here we present the first measurements of the FFR using MEG, and locate its origins. Our principal aim was to use MEG to test whether the FFR emerges only from the brainstem nuclei, as is widely held, or whether a cortical origin may also exist. Specifically, we evaluate the hypothesis that at the relatively low fundamental frequency that is typically used in the literature (Supplementary Fig. 1), the FFR reflects a contribution from the auditory cortex (AC) in addition to contributions from subcortical nuclei^6^. We simultaneously collected MEG and EEG data to first establish that the MEG equivalent of the EEG–FFR can be observed. Using several complementary methods to examine localization, temporal order and behavioural relevance that rely on different assumptions, we present evidence in support of a contribution from the AC, with a right-hemisphere bias. Finally, to test the validity of the conclusions regarding cortical origins of the FFR, we demonstrate that the amplitude of the right AC component of the FFR correlates with behavioural variables relevant for musical processing.

## Results

### MEG equivalent of the scalp-recorded EEG–ABR

**Figure 1:**
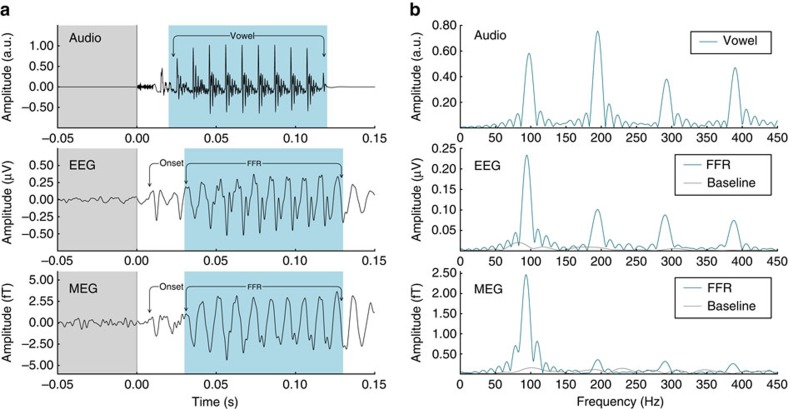
Audio trace, EEG–ABR and single-channel MEG–ABR grand averages. A single MEG channel was selected by maximum correlation with the EEG channel. (**a**) Time course of speech stimulus (syllable: /da/) and EEG/MEG responses, showing that the onset response and the FFR commonly studied with EEG are preserved in the single-channel MEG–ABR. The prestimulus baseline (−50 to 0 ms) and the frequency-following response (FFR) periods (30 to 130 ms) are marked in grey and blue, respectively, for the EEG and MEG responses. (**b**) Corresponding spectra of the periodic portion of the audio signal and the FFR of the responses are shown in blue. Baseline spectra are in grey (*n*=20).

To ensure that the observed MEG response represents the magnetic counterpart of the EEG–ABR, we compared the time and frequency domain averages of the EEG–ABR with the MEG sensor trace that maximally correlated with it (Fig. 1a; mean Pearson's *r*=0.59, s.d.=0.13). Both the onset response and FFR were visible in the EEG and MEG recordings. In the frequency domain (Fig. 1b), both EEG and MEG show clear peaks at the fundamental frequency of the stimulus (f0) and smaller harmonic peaks (integer multiples of the f0). The peak amplitude of the f0 and the signal-to-noise ratio (SNR) between FFR and baseline correlated across EEG and MEG measures (Spearman correlation, *r*=0.43, *P=0.03; *r*=0.50, *P=0.01; Supplementary Fig. 3), demonstrating shared information. To exclude the possibility of electromagnetic contamination from stimulation, we replicated a previous finding that as stimulus amplitude decreases, the latency of the ABR increases^43^ (Supplementary Fig. 4).

Harmonics and other spectral information at frequencies higher than f0 are targets of ABR studies for their importance in speech and musical sounds^44^. Though they were not the focus of this work, we were able to observe that these frequencies were also present in the MEG data (Supplementary Fig. 5); they are best observed in a single-channel grand average by selecting sensors that are most highly correlated with a high-pass-filtered EEG data, as opposed to selecting those sensors which most highly correlate with the time course of the EEG (Fig. 1).

### Region of interest spectra from distributed source modelling

**Figure 2:**
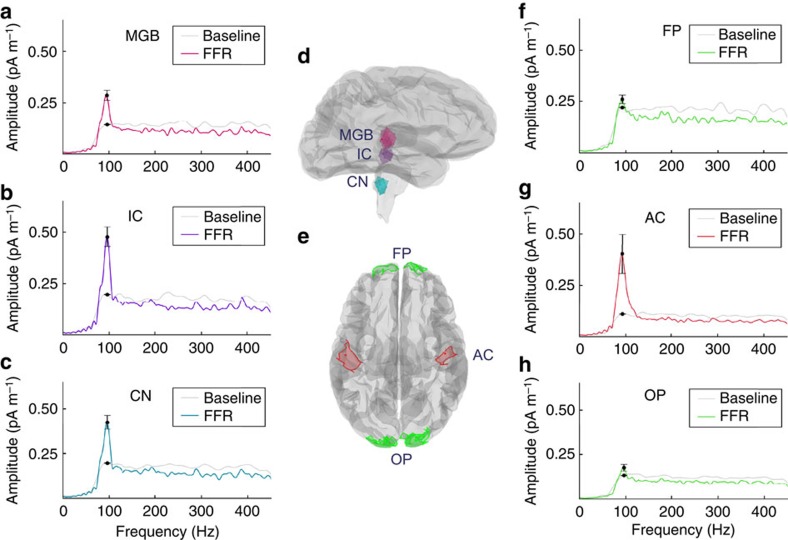
Region of interest (ROI) locations and their spectra during FFR and baseline showing a large contribution at the fundamental frequency for brainstem nuclei and auditory cortex in the MEG data. (**a**–**c**) show the spectra of the three subcortical ROIs. (**d**) Single-subject illustration of the locations of subcortical ROIs; and (**e**) the locations of the cortical ROIs including two control regions in the frontal and occipital poles. (**f**–**h**) Spectra of the cortical ROIs. Error bars indicate s.e. of the mean. All results are averaged across left and right ROIs and across subjects (*n*=20). AC, auditory cortex; CN, cochlear nucleus; IC, inferior colliculus; MGB, medial geniculate nucleus; controls: FP, frontal pole; OP, occipital pole.

To separate the contributions of subcortical and potential cortical FFR sources, we used minimum-norm estimate (MNE) modelling of head tissues based on individual anatomy. Data from bilateral pairs of auditory system region of interests (ROIs; AC; medial geniculate nucleus, MGB; inferior colliculus, IC; and cochlear nucleus, CN), plus two control ROIs not expected to show auditory responses (frontal pole and occipital pole) were extracted, and their power spectrum density computed. This analysis showed a strong peak at f0 from the AC ROI that was significantly greater than residual signal observed in control regions; this was also the case for all the subcortical auditory ROIs (Wilcoxon-matched pairs test, *Z*=−3.92, *P<0.001 for each; Fig. 2). The magnitude of these latter components is similar to that of the cortical sources, indicating that MEG is sensitive both to deep and superficial sources. These results were not strongly sensitive to the MNE depth-weighting parameter, and all parameter values lead to f0 peaks that were significantly above baseline levels (Wilcoxon signed-rank test, *Z*=−3.92, *P<0.0001 for each comparison).

### Comparison of forward projection of the AC signal with data

**Figure 3:**
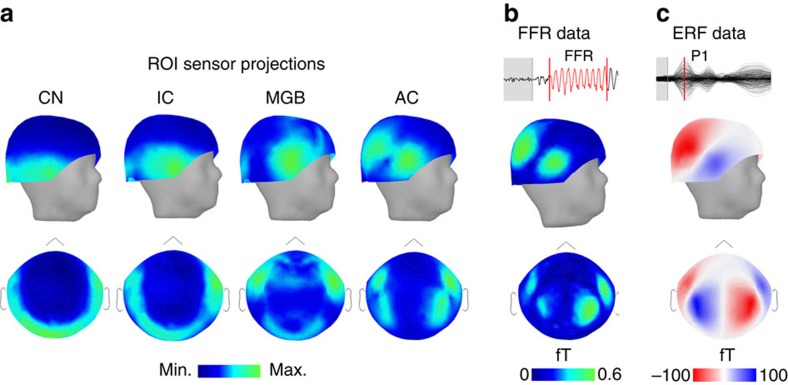
Comparison of FFR topography to that of sensor projected source activity from anatomical ROIs and the cortical ERF. All topographies are averaged across subjects (*n*=20). (**a**) Sensor distribution from bilateral region of interest (ROI) sources, in three-dimensional (side view) and two-dimensional (top view) sensor space. For each ROI, the colour map is scaled to minimum and maximum signal strength to show the distribution of sensor sensitivities to each source. (**b**) The topography of fundamental frequency magnitude in the FFR. (**c**) ERF topography at P1 (∼73 ms; independent data set) was significantly correlated with FFR topography for all subjects. The absolute value is taken before correlation with the FFR topography.

The expected distribution of information from each left–right pair of auditory system ROIs as revealed using forward projection through the MEG head model is illustrated in Fig. 3a; these are used as ROI topography templates in the following analysis, and can also be used to compare the relative strengths of each ROI's contribution to and correlation with the recorded data. The result showed the strongest response from AC, followed by the CN, medial geniculate and IC (Supplementary Fig. 6a,b).

### Model-free comparison of FFR and known AC MEG topography

To obtain an estimate of cortical involvement that was free from modelling assumptions, we identified the first large peak of the evoked response field (ERF) in a separate long inter-stimlus interval data set (P1; ∼74 ms; Fig. 3c), which is of known cortical origin^45^. We then compared the distribution of f0 signal across sensors during the FFR (30–130 ms post stimulus onset); with that of the ERF signal. The two bore a striking resemblance (Fig. 3b,c) and were significantly correlated in all subjects (mean *r*=0.50, s.d.=0.24), supporting the existence of a cortical contribution to the FFR.

### Respective timings of cortical and subcortical contributions

**Figure 4:**
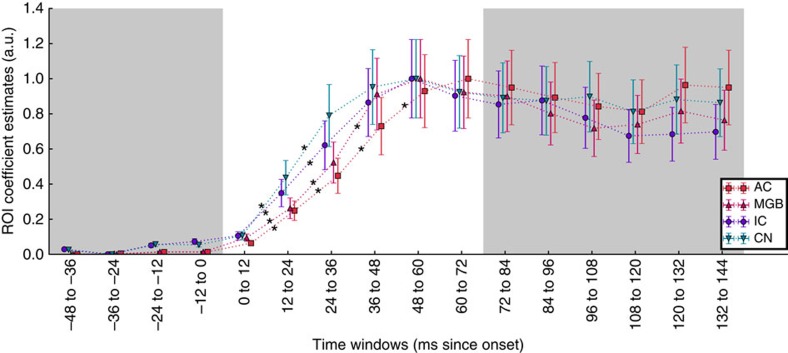
Respective timings of the four putative sources. The explanatory power (coefficient estimates) of ROIs over successive 12 ms windows from sound onset to 72 ms. * Indicates significant increases between successive windows; CN and IC increase only up to 24–36 ms window, whereas MGB increases up to the 36–48 ms window and AC increases up to the 48–60 ms window; contributions from each structure peak successively (*n*=20). Results are scaled between 0 and 1 to better visualize the relative time courses of ROIs, and grey areas indicate the prestimulus period and the stable FFR period, which were not the subject of the statistical tests. Error bars show s.e.m. and are slightly offset for visibility. (MGB, medial geniculate body; IC, inferior colliculus; CN, cochlear nucleus; AC, auditory cortex.

To confirm that the cortical component showed a physiologically plausible delay with respect to components from earlier structures, we calculated for each of the four pairs of ROIs the root mean square (r.m.s.) levels of time courses of coefficient estimates over successive windows (see Methods for details), using the ROI topography templates as linear regressors. Coefficient estimate time courses can be interpreted as the ability of each ROI to explain the observed signal over time (Fig. 4). The explanatory power of the CN increased over the first two windows (0–12 ms, 12–24 ms post stimulus onset), but further increases were not distinguishable from chance (see Table 1 for statistics). Similarly, the IC increased significantly over the first two windows, and not thereafter. The MGB increased significantly up to and including the third window (24–36 ms). The AC increased significantly up to and including the fourth window (36–48 ms) before plateauing. These statistics suggest that the respective onsets of contribution from each structure to the scalp signal are spread out over time, in an order corresponding to physiological expectations.

The explanatory power of the ROI (coefficient estimates) fluctuates over time, with maximum values at time points that represent the FFR peaks and zero values at FFR nodes, when the ROI templates do not describe the signal. If sources cannot be separated, the maximum cross-correlation between the two time courses would be found at latencies close to zero milliseconds. Cross-correlation analysis of the oscillation within the coefficient estimate time courses of successive ROIs (for example, CN, IC, MGB and AC) showed a median time delay of 5.5 ms (s.d.=0.5) between the CN and IC (see Supplementary Fig. 7, bottom). The CN and IC time courses were separable in all subjects, supporting the ability of MEG to resolve signals from different brainstem structures. The cumulative median time delay between CN and MGB was 10.8 ms (s.d.=2.7), and between CN and AC was 14.6 ms (s.d.=4.6). In each of these steps, several subjects had values close to zero, indicating that cross-correlation analysis failed to show a clear separation of the signals (this is visible as data points close to 5 ms in Supplementary Fig. 7, middle; and close to 5 and 10 ms in Supplementary Fig. 7, top); however, a cluster of subjects with clearly separable signals for each step show a CN to AC delay of about 16 ms. These data further support biologically plausible delays between information coming from successive structures in the auditory system, but because only positive cross-correlation values were accepted and because time of later stages is cumulative, it is not meaningful to statistically evaluate successive delay increases; they are therefore provided for descriptive purposes. These data also validate the use of MEG for deep auditory brainstem sources, as deep sources can be separated from one another.

### Whole-brain volume modelling

**Figure 5:**
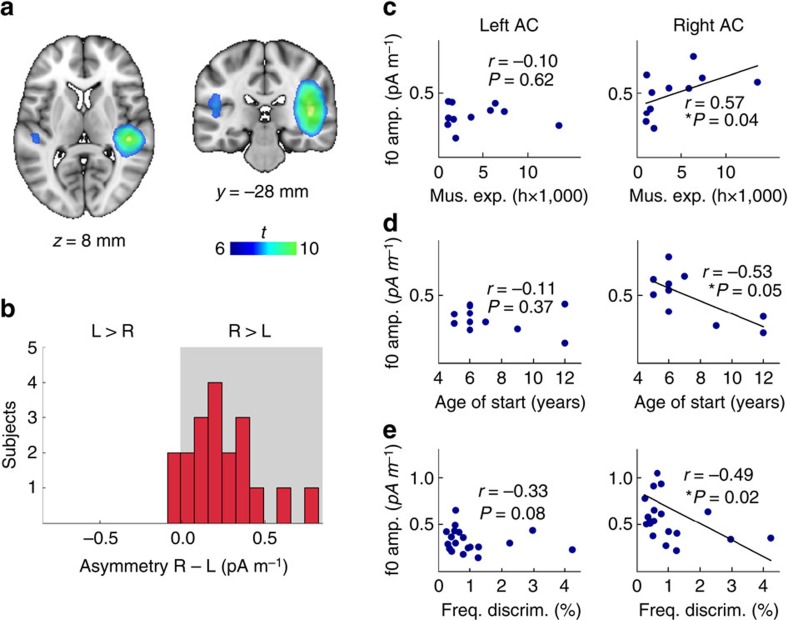
Cortical asymmetry and behavioural correlations. (**a**) Whole-brain MEG source results from a minimum-norm estimate (MNE) volume model (*t*-statistic parametric map, shown in blue–yellow scale) superimposed on the 1-mm MNI152 standard template in stereotaxic space. ABR–FFR signal strength was greater than baseline in two clusters centred on the auditory cortex. Clusters are significant applying cluster-corrected thresholds to control family-wise error rate (*t*>2.3, *P*<0.05); *t*>6 for visualization purposes. (**b**) Distribution of left–right amplitude differences in the auditory cortex ROIs for each individual using the mixed surface-volume MNE model (Fig. 2e) shows strong right-sided asymmetry (*n*=20). Correlations between behavioural variables and f0 amplitude in left and right ROIs: (**c**) training hours in musicians; more training correlated with stronger f0 in the right AC only (*n*=11). (**d**) Age training started in musicians; earlier start ages were correlated with stronger f0 representation in the right AC. (**e**) Fine pitch discrimination (*n*=20); finer pitch discrimination correlated with stronger f0 in the right AC. A non-significant trend is present in the left AC.

To verify that cortical sources were spatially specific to the AC (rather than other cortical regions), we used whole-brain volume source models and permutation testing to produce a statistical parametric map (Fig. 5a). This method is also dependent upon an MNE model, but is based on a whole-brain volume (as opposed to a combined surface and volume) and therefore allows for co-registration between subjects and group analysis in source space. It is not well suited to measuring brainstem nuclei sources because of the volume model used, and particularly because the brainstem does not align well across the subjects. The cortical contribution to the FFR was localized in two areas, one in each hemisphere, centred over Heschl's sulcus in the AC. No other significant or sub-threshold clusters were detected in cortical regions, confirming that the FFR cortical sources arise from auditory regions and not other cortical locations.

### Asymmetry of the cortical FFR and behavioural relevance

Inspection of the data revealed a large hemispheric asymmetry. To test for the significance of this asymmetry in FFR magnitude, we used f0 signal strength derived from the AC ROIs in the more anatomically accurate mixed model described in Fig. 2. The mean f0 amplitude in the left AC was 0.39 pA m^−1^ (s.d.=0.27), whereas in the right AC the mean was 0.68 pA m^−1^ (s.d.=0.50). The right AC was significantly stronger than the left (Wilcoxon signed-rank test, *Z*=−3.62, *P<0.001; Fig. 5b). This asymmetry was not present in the first peak of the ERF data (left AC mean=2.82 pA m^−1^ (s.d.=1.80); right AC mean=3.08 pA m^−1^ (s.d.=1.69); Wilcoxon signed-rank test, *Z*=−0.04, *P*=0.97; Supplementary Fig. 8). This finding indicates that the asymmetry is specific to the FFR and not a general feature of the MEG response.

To further validate our claim of a cortical contribution, we evaluate whether the asymmetry in the signal attributed to the cortex has meaningful relationships to behavioural variables in a manner that is consistent with literature on cortical functional specialization. We confirmed the hypothesis that f0 strength of the FFR is positively correlated with cumulative hours of musical experience and negatively correlated with age of musical training onset in the right, but not left AC in a subset of eleven subjects who reported musical experience (Fig. 5c,d). We also observed a significant correlation with pitch discrimination thresholds across the entire sample (Fig. 5e). However, there was no correlation with a simple melody discrimination task (left AC: Spearman's *r*=0.11, *P*=0.33; right AC: *r*=0.12, *P*=0.32). When the FFR was computed from the simultaneously recorded EEG, the relationships between musical experience and fine pitch discrimination thresholds were not statistically significant (reported for completeness—EEG–f0 versus hours of musical training: *r*=0.27, *P*=0.21; age of start: *r*=0.03, *P*=0.53; fine pitch discrimination: *r*=−0.25, *P*=0.14).

## Discussion

Our results show that a right-lateralized FFR originates in the cortex and is strongly represented in the MEG equivalent of the EEG–ABR. This conclusion is supported by six lines of converging evidence: (a) distributed source models show a strong signal in the auditory cortices; (b) a forward projection of the signal from AC is correlated with recorded data; (c) the topography of FFR power correlates with the topography of the cortical ERF; (d) the temporal dynamics of each ROI peak in succession at biologically plausible latencies; (e) cortical components are localized to Heschl's sulcus bilaterally in a whole-brain analysis; and (f) the magnitude of the response at the fundamental frequency in the right AC is significantly related to musicianship and pitch perception. In addition to the cortical contribution, our data show the expected contributions from all other major subcortical auditory nuclei.

In measuring the MEG equivalent of the EEG–ABR, we expect to be measuring partially overlapping and partially distinct aspects of the same underlying phenomenon. This is because MEG is insensitive to radial sources, while EEG may reflect both radial and tangential sources, and MEG is comparatively less sensitive to deep sources^36^. Both the onset response and FFR are clearly visible in the MEG–ABR (Fig. 1); the peak amplitude of the f0 and the SNR between FFR and baseline correlate across EEG and MEG (Supplementary Fig. 3), and the MEG–ABR demonstrates a similar relationship as its EEG counterpart between response latency and stimulus amplitude. These findings confirm the identity of the recorded signal as the MEG equivalent of the EEG–ABR, as a basis for further comparison.

Using distributed source models based on individual anatomy, which allow us to disentangle simultaneously active sources on the basis of the amplitude and sensor distribution of recorded data, we showed that the signal attributed to each cortical and subcortical auditory region is greater than residual signal in control areas (Fig. 2). Forward projection of each bilateral pair of ROIs shows how information modelled as originating in the ROIs would be distributed over sensors, taking into account the depth, strength and orientation of the sources (Fig. 3a). The topographic distribution of the recorded FFR data resembles and is significantly correlated with the AC ROI projection topography (Fig. 3a,b), with smaller correlations with subcortical structure topographies (Supplementary Fig. 6a). Similarly, the AC accounted for the highest relative percentage of signal within the MEG data (Supplementary Fig. 6b). It should not be assumed that these percentages hold for the EEG–FFR, as EEG and MEG recordings likely represent different relative weighting of these signal generators. These analyses are based on an anatomically based MNE source model, with default parameters. MEG imaging of deep sources is an area of active development, and a full account of the effects of modelling parameters on deep sources is not yet available; however, we found that the main results are not strongly sensitive to variations in depth-weighting. While we find clear evidence for a cortical source, localization of MEG sources is always subject to caution; hence, additional validation with techniques that have more direct ability to localize sources of neural activity, such as functional magnetic resonance imaging (MRI) and intracranial recordings, will be necessary in future to confirm our principal conclusion.

Although it is unlikely to affect the cortical results, for which distributed source models such as the MNE have been developed and validated^36^,^46^, we nonetheless addressed the existence of a cortical FFR contribution without reference to the source model by comparing the topography of the FFR power (Fig. 3b) with the topography of the cortical ERF at a peak with a known cortical origin (Fig. 3c). This data set was recorded in the same subjects in an independent run, which was pre-processed in accordance with standard practice to isolate subcortical and cortical components by frequency band^1^. This analysis further supports the conclusion of a cortical source because its distribution was significantly correlated with the known cortical response in the ERF. Because this analysis takes place in sensor space, this result cannot be attributed to biases of the source model.

We evaluated whether the temporal dynamics of each ROI peak in succession at biologically plausible latencies. The typical stimulus–response delay when measuring FFRs is 6–10 ms and reflects the transmission delay between the ear and the rostral brainstem structures^6^, whereas a response from the AC is not expected until at least 13 ms^40^. In our single-channel time-domain average, a delay that might account for the neural transmission to the cortex is not evident. We obtained time courses of regression coefficient estimates for each of the ROI topographic templates, and used these to show that the values of each ROI, which could be considered explanatory power, peaked in the expected succession starting with subcortical CN and IC, followed by the MGB, and finally the AC (Table 1, Fig. 4). This ordering is corroborated by an exploratory analysis of between-ROI latencies derived from a cross-correlation analysis using the oscillation within the same data, which shows that the cumulative response time of successive ROIs is in the order of 14 ms to the cortex.

The results from an analysis using whole-brain source models showed that the cortical contribution was centred over the primary AC in both hemispheres (Fig. 5a), and in agreement with the sensor space data (Fig. 3b), the signal appears to be strongly right lateralized. We confirmed that signal attributed to the right AC ROI was significantly stronger than the left AC ROI across the subjects (Fig. 4b), while the first peak in the cortical ERF was not lateralized (Supplementary Fig. 7). This dissociation supports a genuine functional asymmetry in FFR strength rather than an artifact of asymmetric cortical folding^47^. Furthermore, as a means of validating the functional significance of the asymmetry, we showed a relationship between the measures of musicianship and of fine pitch discrimination skills with the strength of signal originating in the right AC (Fig. 5c–e). This analysis serves to support the existence of a cortical contribution (rather than rule out a subcortical enhancement), as it is unlikely that a subcortically generated signal misattributed to the cortex would show a clear lateralization and relationship to behaviour.

These behavioural correlations suggest that the asymmetric contribution likely is relevant to the processing of pitch information. This conclusion is in agreement with longstanding evidence that the right AC is relatively specialized for aspects of tonal processing, coming from studies of patients with tonal processing disorders^48^,^49^, patterns of functional activation^50^,^51^,^52^ and connectivity^53^, and brain stimulation^54^,^55^. The new contribution here is that the fine-grained representation of periodicity reflected in the FFR is more strongly associated with processes in the right AC, which in turn may be the underlying reason why many phenomena previously described may also demonstrate asymmetry. An important new question emerging from these findings is whether periodicity analysis within brainstem structures is influenced by efferent but asymmetric cortical influences, and/or whether relevant computations at the brainstem level^56^ contribute to the cortical asymmetry.

Because EEG–ABRs are measured using montages that span the AC and EEG is sensitive to both radial and tangential sources^36^, our results suggest that the underlying neural activity is also captured to some degree in EEG recordings. The EEG studies described in the introduction have typically interpreted the FFR as being of purely subcortical origin. It has also been argued explicitly^6^ that a cortical contribution is unlikely because of several reasons: there is a lack of a repetition suppression effect in the FFR; the FFR may reflect a succession of subcortical onset responses; the ABR reaches maturity in early childhood, whereas cortical signals are slower to mature; and neurons within the AC have an upper limit to phase-locking of about 100 Hz (ref. 57). New evidence has come to light that should be considered when evaluating each of these arguments: both response inhibition and response enhancement were reported (and related to learning speed)^58^; the FFR cannot be fully accounted for by modelling it as a series of overlapping onset responses^11^; and the FFR is now known to change throughout childhood stabilizing in late teenage-hood^21^. Recent preliminary intracranial recording data also showed an FFR recorded directly from the human AC at ∼200 Hz (ref. 59). These findings are compatible with our conclusion that a cortical source coexists with subcortical sources.

We selected a stimulus to reflect the most commonly used features in the extant literature, in order that our findings would be relevant in interpreting the conclusions drawn from these studies. In that context, we used a relatively low-frequency f0 (98 Hz), since this value is close to that of a large number of studies (Supplementary Fig. 1). This frequency is likely within the phase-locking capabilities of single neurons in the cortex^57^, thus making our claim of a cortical origin for the FFR at this frequency physiologically plausible; however, single-unit firing frequencies may not be an accurate way to estimate the upper limit of phase-locking of populations of neurons in the cortex. Our pattern of results might be accounted for by the existence of a population of less numerous or less-synchronized neurons near to primary AC that is distinct from the generators of typical auditory evoked potentials, a suggestion that is also supported by the difference in symmetry between the FFR and ERF signals. It nonetheless seems likely that the cortical contribution to the FFR would decrease at higher frequencies, and perhaps be absent altogether at very high frequencies. This question remains open for future work, which however can only be undertaken once a cortical contribution at lower frequencies is fully documented, which was our goal here.

ABR results are likely influenced by cortical activity to different degrees. ABR onset responses, which occur before neural transition time to cortex has elapsed, logically cannot be influenced by a cortical response and are therefore unaffected by this conclusion. ABR–FFR measures, including f0 frequency strength, may be affected according to the stimulus frequency as discussed above, and the duration of the stimulus. The results described in Fig. 4 suggest that shorter duration FFRs (<40 ms, for example, Tierney *et al*.^24^) may include less cortical contribution than longer ones (>80 ms). Timing and latency of peaks embedded in the FFR may also not be immune to cortical influence, because constructive interference of cortical components in the compound wave can influence wave peaks' latency and amplitude. The strength and distribution of harmonic frequencies (Supplementary Fig. 5) as compared with the ROI topographies of the f0 (Fig. 3a) suggest that the harmonics originate in subcortical regions. This should not be taken as indicating a cutoff of phase-locking in the cortex between 100 and 200 Hz; however, because the stimulus' spectral content and envelope show a behavioural dissociation^4^,^44^ and may be represented in different locations in the brain.

While FFR studies have uncovered important phenomena in a wide range of fields, we propose that their exclusive attribution to subcortical generators should be re-examined in light of our findings. MEG may provide the technical means to do so. The relationship with behavioural measures illustrated in Fig. 5c–e can serve to illustrate how MEG might be useful. The previous FFR studies have demonstrated a correlation between EEG–FFR amplitude and musical experience, age at which musical training began and pitch discrimination performance; these phenomena were attributed to subcortical enhancements^13^,^14^. Our cortical correlations certainly do not imply that there is no subcortical enhancement. In fact, it is quite possible that we observe enhancements in the cortical signal because the cortex has received better ‘pre-processed' information from lower centres. Current analytical tools do not allow us to untangle these relationships, but using dedicated experimental paradigms that contrast groups and conditions similar to those used in existing work, MEG–FFR may allow us to observe the relative changes in activity of different nuclei of the auditory system, while they are interacting with one another.

We support an emerging viewpoint in the literature that the FFR component of the ABR represents an integrated response of the entire auditory system^10^. The strong bidirectional anatomical connections between higher and lower nuclei in the auditory system^3^ suggest that a great deal of functional interaction occurs during the normal processing of sound. Using MEG to observe the interaction of these components as they contribute to the scalp-recorded composite FFR may complement existing EEG methods, and play a supporting role in clarifying specific research questions about phenomena first observed with EEG.

By demonstrating that the FFR consists of cortical and subcortical components, and offering a means by which these contributions can be measured, we hope to facilitate a deeper understanding of how the auditory system processes information, changes with experience, and is affected by pathology. With improved spatial information, fine temporal resolution and powerful analysis methods, MEG will be an excellent tool to disentangle the complex interactions between cortical and subcortical auditory structures in health and disease across the lifespan. This may in turn facilitate targeted interventions and treatments for health problems related to auditory processing.

## Methods

### Participants

Twenty-two neurologically healthy young adults who had previously undergone a T1 structural MRI as part of unrelated studies were recruited. Two were later excluded; one for excessive muscle activity and movement during recording, and the other due to the presence of abnormal neurogenic spiking activity over the temporal lobes. The mean age of the remaining participants was 25.7 years (s.d.=4.2), 12 were female and all were right handed, had normal or corrected-to-normal vision and had no history of neurological disorders. Sample size was selected to be able to show that the MEG–ABR can be measured consistently in sample sizes comparable to those used in previous EEG–ABR work, and to be sufficiently large to investigate behavioural relationships. Informed consent was obtained and all experimental procedures were approved by the local ethics committee (Montreal Neurological Institute Research Ethics Board).

### Study design

Subjects were first screened for neurological conditions and information about their musical experience collected via an online version of the Montreal Music History Questionnaire.^60^ Before the MEG session, audiometry was collected to ensure normal hearing. Behavioural data were collected using computerized tasks (∼45 min). Subjects were prepared for EEG, and instructed to relax and keep still, followed by an MEG recording of 1–1.5 h.

### Audiometry

An audiometric test was administered to control for basic hearing function using a Maico MA 728 audiometer (Maico, Minneapolis, MN, USA). Pure-tone hearing thresholds were assessed in each ear for 500, 1,000, 2,000, 3,000, 4,000, 6,000 and 8,000 Hz. All but three participants had ≤15 dB hearing level (HL) pure-tone thresholds. The remaining three had slightly elevated thresholds at one or more higher test frequencies, but ≤30 dB HL over the whole range and≤25 dB HL for frequencies ≤4,000 Hz—these subjects were included in the study as the 80 dB sound pressure level (SPL) sound presentation was well above threshold.

### Behavioural tasks

Fine pitch discrimination thresholds were measured using a two-interval forced-choice task and a two-down one-up rule to estimate the threshold at 79% correct point on the psychometric curve^61^. On each trial, two 250 ms pure sine tones were presented, separated by 600 ms of silence. In randomized order, one of the two tones was a 500 Hz reference pitch, and the other was higher by a percentage that started at 7 and was reduced by 1.25 after two correct responses or increased by 1.25 after an incorrect response. The task stopped after 15 reversals, and the geometric mean of the last eight trials was recorded. The task was repeated five times, and the scores averaged. Subjects also performed a simple melody discrimination task^62^, in which they were asked to judge whether two unfamiliar melodies in the western major scale were the same or different. Melodies were made up of 5–13 complex tones (each of which was 320 ms in duration) at a tempo of 93.75 beats per minute, and used pitches whose f0 was between C4 and E6. On half the trials, the pitch of one note was altered by up to ±5semitones, while maintaining the key and melodic contour of the melody. Data were also collected for several other tasks not reported here.

### Subject preparation

A single-channel (Cz; 10–20 International System) EEG montage was applied with a forehead ground and earlobe references^1^. Bipolar EOG electrodes around the eyes and ECG electrodes on the chest were applied for later use in detecting corresponding artefacts in the MEG traces. Head shape and the location of head position indicator coils were digitized (Polhemus Isotrak, Polhemus Inc., VT, USA) for co-registration of MEG with anatomical T1-weighted MRI.

### Data acquisition

Two hundred and seventy channels of MEG (axial gradiometers), one channel of EEG data, EOG and ECG, and one audio channel were simultaneously acquired using a CTF MEG System and its in-built EEG system (Omega 275, CTF Systems Inc.). All data were sampled at 12 kHz. To control for attention and reduce fidgeting, a silent wildlife documentary was projected onto a screen at a comfortable distance from the subject's face (Yellowstone: Battle for Life, BBC, 2009). The video image was kept small (∼15-cm wide at arm's length) to minimize involuntary saccades. We collected data from two additional audio stimulus amplitudes (70 and 60 dB SPL) from one subject as a control to confirm a biophysiologically plausible relationship between stimulus amplitude and response latency; blocks of quieter stimuli were interleaved pseudorandomly with the 80 dB runs.

### Stimulus presentation

The stimulus was selected as most appropriate to meet two goals: to be relevant for the interpretation of previous work (Supplementary Fig. 1); and to maximize the likeliness of obtaining a clear MEG–FFR^1^, as it has not previously been measured. The stimulus was a 120-ms synthesized speech syllable (/da/) with a fundamental frequency in the sustained vowel portion of 98 Hz. This syllable is favoured by many ABR researchers for its acoustic properties, ecological validity in speech (human speech f0: 80–400 Hz) and ability to produce robust ABRs, including both a clear onset and FFRs in most subjects^1^.

The stimulus was presented binaurally at 80 dB SPL, ∼14,000 times in alternating polarity, through Etymotic ER-3A insert earphones with foam tips (Etymotic Research). For five subjects, ∼11,000 epochs were collected due to time constraints. Stimulus onset synchrony (SOA) was randomly selected between 195 and 205 ms from a normal distribution. The recording session was divided into five runs of ∼7 min (∼1500 stimuli of each polarity), which were separated by silent pauses of several minutes to allow the subject to rest and to accommodate data transfer. A separate run was collected of ∼600 stimulus repetitions spaced ∼500 ms apart, to record the slower cortical responses.

The audio signal was split and recorded as a channel in the data such that each stimulus onset could be precisely determined. To decrease the possibility of electromagnetic contamination of the data from the signal transducer, ∼1.5-m air tubes between the ear and the transducer were used such that the transducer could be tucked into a shielded cavity on the floor (>1 m from the MEG gantry, behind and to the left of the subject).

### EEG and MEG preprocessing

Data analysis was performed with Brainstorm^63^ and using custom Matlab scripts (The Mathworks Inc., MA, USA). Eye blink and heart beat artefacts were removed from MEG data using Brainstorm's in-built source signal projection algorithm^64^,^65^, using the recommended procedure: projectors were removed when they captured at least 12% of the signal and the topography of the components matched those of ocular or cardiac origin upon visual inspection.

Sound onsets were marked by a custom threshold-based algorithm that detected onsets in the simultaneously recorded audio trace. After band pass filtering (80–2000 Hz) and epoching (−50 to 150 ms relative to stimulus onset), simple threshold-based artefact rejection was applied of ±35 μV on EEG channels and ±1,000 fT on MEG channels. On average, 97.5% of epochs were kept (s.d.=3.2%). Subject averages were created by first averaging epochs of each polarity and then summing negative and positive polarity averages. As few subjects' ABRs contained power at harmonics above the fourth (392 Hz), we further low-pass-filtered time-series averages below 450 Hz. The cortical run was processed with band pass filters set to 2–40 Hz, epoched from −50 to 450 ms and epochs were averaged. Sound took ∼4.5 ms to reach the ear; this was considered in analyses involving measures of latency.

### MEG–FFR signal quality

To ensure that we were able to consistently observe a clear MEG–ABR, we selected the channel with the greatest time-domain SNR per subject, calculated as the ratio of r.m.s. signal amplitude during the FFR period (30 to 130 ms post stimulus onset) to the baseline period (−50 to 0 ms). The SNR for each subject and each modality was confirmed to be >1.5, which is often used as an exclusion criterion for signal quality^1^. Previous work used 3–5 times more epochs to obtain MEG click responses^40^ than are normally used for ABR studies^1^. To record MEG–FFR for the first time, we also used a high number of stimulus repetitions, but this appears not to be strictly necessary for studying f0 amplitude; SNRs>1.5 was possible for most subjects when 4,000 epochs or more were included in the average.

### MEG equivalent of the scalp-recorded EEG–ABR

The potential benefits of developing MEG to study the ABR lie in methods that use multiple channels; however, because EEG is most often measured using a single channel, we must select a single MEG channel to demonstrate successful recording of the MEG–ABR as a basis for more advanced analyses. If we have captured the MEG–ABR, it should display the two important features of the ABR to periodic sound: the onset response and FFR. Because the ABR is defined by features as measured using EEG, we selected the MEG channel that was most correlated with the EEG channel per subject (MRO24 (four subjects), MRO34 (two subjects), MRO14 (two subjects), MLT24, MRF67, MRO22, MLF55, MLT42, MRP45, MLT34, MLT43, MLT53, MRO32, MRO23′, MRT15 and MLT23; see Supplementary Figure 2 for channel locations) and calculated a grand average. This choice yields the clearest onset response; however, note that selecting the channel with the largest SNR or the strongest signal overall give very similar results. Selecting the MEG channels that best correlate with EEG–ABRs that have been filtered to exclude f0 can be used to emphasize the harmonics (selected channels by subject: MRP23 (two subjects), MRO22 (two subjects), MRO32 (two subjects), MRO33, MRO13, MRO34, MLT24, MRO24, MLT31, MLT31, MLT42, MLT42, MLT43, MLT43, MLT51, MLF56 and MLC12). A Bonferroni correction for multiple comparisons was applied to the critical value over the 270 MEG channels for each subject (0.05/270).

Though the EEG and MEG–FFR may reflect different combinations of information from different sources, they should also share information. Because magnetic fields decrease rapidly with distance, small differences in head shape, and positioning under the helmet and movements could lead to suboptimal MEG measures of signal strength with respect to the scalp-recorded EEG signal. Nonetheless, to further support equivalence of the EEG and MEG signals, we computed Spearman's correlation on the SNRs of the EEG and MEG channels, and on the amplitude of fundamental frequency (Supplementary Fig. 3; here and elsewhere non-parametric statistics were used when data were not normally distributed).

The amplitude of the f0 component of the signal was selected using an automatic script, from each signal's spectrum, which was obtained by first windowing the signal (5 ms raised cosine ramp), zero padding to 1 s to enable a 1 Hz frequency resolution, with subsequent fast Fourier transform, and rescaling by the proportion of signal length to zero padding.

To guard against the possibility that the MEG–ABR signal is an electromagnetic artefact of stimulus generation, we replicated the finding that FFR latency increases with decreasing stimulus sound level using EEG^43^. We assessed the stimulus–response latencies (maximum correlation between 5 and 15 ms) using cross-correlation from averages derived from blocks of 80, 70 and 60 dB SPL stimulus intensities, which were presented in a randomized order. To determine if these represent statistically significant differences between intensity conditions within each modality, we computed bootstrap statistics. Sub-averages were calculated by resampling 3,700 epochs from the original pool of epochs with replacement, summing opposite polarities and obtaining the stimulus–response lag as above. The s.e. of the stimulus–response lag was computed as the s.d. of 10,000 iterations. Cross-correlation analysis of an oscillatory signal selects maxima at multiples of the stimulus' period and correlations drop off sharply as shift increases, which creates a non-normal distribution. To statistically evaluate the results, we used a Kruskal–Wallis test, with Mann–Whitney *U* tests between successive stimulus amplitudes as planned *post hoc* comparisons; non-parametric statistics were used as data were not normally distributed.

### Region of interest spectra from distributed source modelling

Distributed source models estimate the amplitude of a large set of dipoles on the cortical surface or within the entire brain volume^66^. These models are better able to map activity originating at multiple generator sites^36^, but they must be constrained by spatial priors.

FreeSurfer^67^,^68^ was used to prepare cortical surfaces and automatically segment subcortical structures from each subject's T1-weighted anatomical MRI scan. The results were imported into Brainstorm^63^, and the brainstem and thalamic structures were combined with the cortex surface to form the image support of MEG distributed sources: the mixed surface/volume model included a triangulation of the cortical surface (∼15,000 vertices), and brainstem and thalamus as a three-dimensional dipole grid (∼18,000 points). An overlapping-sphere head model was computed for each run; this forward model explains how an electric current flowing in the brain would be recorded at the level of the sensors, with fair accuracy^63^. A noise covariance matrix was computed from 1-min empty-room recordings taken before each session. The inverse imaging model estimates the distribution of brain currents that account for data recorded at the sensors. We computed the MNE source distribution with unconstrained source orientations for each run using Brainstorm default parameters. The MNE source model is simple, robust to noise and model approximations, and very frequently used in literature^64^. Source models for each run were averaged within subject.

ROIs were used to retrieve the signal attributed to specific brain regions during the FFR period (30–130 ms post stimulus onset; Fig. 2d,e). We combined the regions identified as transverse temporal gyrus and transverse temporal sulcus as the AC (L: 7.40 cm^2^ (s.d.=1.18); R: 5.5 cm^2^ (s.d.=1.04)). Two control ROIs were selected in regions unlikely to participate in sound processing, and located at maximal distance from the target auditory regions: the frontal poles (L: 7.41 cm^2^ (s.d.=1.38); R: 12.47 cm^2^ (s.d.=2.22)) and occipital poles (L: 17.18 cm^2^ (s.d.=1.94); R: 25.62 cm^2^ (s.d.=2.87)). Roughly spherical subcortical volume ROIs were grown from seeds in dipole grid around the right and left cochlear nuclei (estimated with reference to the medullary pontine junction; L: 0.49 cm^3^ (s.d.=0.06); R: 0.48 cm^3^ (s.d.=0.06)) and the right and left IC (identified by contours of the brainstem; L: 0.50 cm^3^ (s.d.=0.04); R: 0.47 cm^3^ (s.d.=0.06)). ROIs in the right and left posterior thalamus meant to capture activity in the medial geniculate bodies were defined—these regions covered approximately the posterior third of the thalamus (L: 1.35 cm^3^ (s.d.=0.06); R: 1.25 cm^3^ (s.d.=0.14)). Subcortical ROIs are each bigger than the structures of interest to maximize the likelihood of capturing signals. We extracted a timeseries of mean amplitude for each ROI and for each of the three orientations in the unconstrained orientation source model. Orientations were summed in the frequency domain (calculated as described above), to yield a single spectrum for each bilateral pair of ROIs during the FFR and during the baseline period (Fig. 2a–c, f–h). To evaluate whether the amplitude peak at f0 was greater in each auditory ROI than in an average of the control regions, we calculated the increase of signal during FFR at f0 over baseline for each bilateral pair and assessed statistical significance using Wilcoxon-matched pairs tests, corrected for multiple comparisons (critical value=0.05/4).

To ensure that the f0 amplitude results from auditory region ROIs are not overly sensitive to the MNE depth-weighting parameter and that the default value (0.5) is appropriate, we recomputed the MNE models using a range of depth-weighting values (0.3–0.9 in steps of 0.2) and assessed whether the results were stable.

### Comparison of forward projection of the AC signal with data

To observe the topography of information from each paired ROI in sensor space, we projected source magnitudes through the MEG forward model to obtain sensor topographical distributions (Fig. 3a); these are used as ROI templates for subsequent analyses. We calculated mean r.m.s. of the simulated data across all channels, and expressed the relative contribution of each ROI as a percentage of the total signal simulated from each for the four auditory ROIs (Supplementary Fig. 6a). We then spatially correlated each ROI topography with the measured FFR amplitude topography (Supplementary Fig. 6b).

### Model-free comparison of FFR and known AC magnetic field topography

The first waves of the ERF measured ∼50–80 ms after stimulus onset are known to originate in the AC^45^; thus, the cortical ERF response overlaps in time with the FFR portion of the stimuli (most of which are 100 ms or more in duration), but is distinguished from it by frequency band (ERF: ∼2–40 Hz, ARB–FFR: 80–2,000 Hz). At sensor level, AC activity produces a bipolar MEG topography over each temporal region (Fig. 3c). For each subject, we extracted the absolute value at each sensor during the peak of the first wave (mean wave latency=74.40 ms, s.d.=13.15 ms) from the cortically processed run. In two subjects, the earliest peaks occurred at 102 and 109 ms, respectively; waveforms at this latency are also thought to originate from the auditory regions^45^ and were used instead.

We reasoned that if there is a cortical component to the FFR, the distribution of power at the fundamental frequency over the sensors should resemble the magnetic field topography of the ERF. We used a Hilbert transform to quantify the magnitude of f0 signal (90–106 Hz) at each sensor. We used Pearson's correlation to assess similarity between the ERF and FFR topographies, that is, for each subject, we measured the correlation between the absolute value of the ERF peak across all sensors with the magnitude of the f0 response across all sensors. Despite that the FFR topography appears more asymmetric than the ERF topography, there is considerable overlap in the distribution of signal across sensors. We corrected for multiple comparisons over subjects (alpha=0.05/20).

### Respective timings of cortical and subcortical contributions

We sought to verify that the AC contribution occurs after the contributions from subcortical sources as a control for physiological plausibility. Assessing the time shift of time-domain signals (as we have used in the stimulus-response lag computation) is not possible here because the unconstrained model yields three time courses per ROI that are not easily recombined into a single time course. As an alternative, we scaled each subjects' paired ROI topography templates between 0 and 1, and entered them as a set of regressors in a series of multiple regressions over time, with the absolute value of the measured data (bandpass <120 Hz to isolate the f0; absolute values are used because the topographies represent magnitude and include only positive values). The result was a time series of coefficient estimates that can be interpreted as representing the relative change in the ability of each set of paired ROIs to explain the measured signal over time. To assess whether the AC signal peaked later than the subcortical signals, we divided the signal into 12 ms bins before and after stimulus onset, calculated r.m.s. levels, and statistically evaluated whether explanatory power increased over the first six successive pairs of windows after sound onset using Wilcoxon signed-rank test (multiple comparisons correction within each ROI: alpha=0.05/6).

The coefficient estimate time series demonstrate an oscillatory component at 2 × f0. To obtain a oscillatory signal at f0 suitable for cross-correlation analysis, we recomputed the coefficient estimate time series without the absolute value. The time a click response takes to reach the AC (∼13 ms) is longer than the period of the 98 Hz f0 (10.3 ms), which results in multimodal cross-correlation distributions. We therefore evaluated time shift between subsequent ROIs, selecting the peak value between 0 and 9 ms for each subject for each pair of ROIs. Importantly, this allows us to include both 0, which would indicate that two sources have not been separated, and a range of delay times that extends above the physiologically possible transmission time between subsequent structures; however, it does mean that the time between more distant structure reflects the cumulative error in each step. Cumulative response delays are included for descriptive purposes rather than statistical evaluation.

### Whole-brain volume modelling

The mixed surface-volume source model is suitable for its anatomical plausibility and sensitivity to deeper sources, but cannot be coregistered across subjects automatically, for technical reasons. To evaluate whether the cortical component originates specifically in the AC, as opposed to other cortical regions, we reanalyzed the data using MNE models wherein the cortex, thalamus and brainstem were modelled as a single volume grid. This approach also allowed us to test hemispheric differences in an unbiased manner. Volume maps were created of the difference in mean-rectified signal amplitude during FFR over baseline for each subject, and were exported as ‘nifty' volumes for analysis in FSL^69^. Difference maps were coregistered to the subject's high-resolution T1 anatomical MRI scan (FLIRT^70^, 6 parameter linear transformation) and then to the 2-mm MNI152 template (12 parameter linear transformation). *Z*-score values within each image were calculated. Permutation testing was used to reveal locations where magnetic signal was greater during FFR than baseline across subjects (non-parametric one-sample *t*-test using ‘randomize' function^71^; 10,000 permutations; Fig. 5a). Family-wise error rate was controlled using threshold-free cluster enhancement as implemented in FSL (*P* <.01).

It has been suggested that part of the EEG–FFR may originate in the auditory nerve^11^. Our source models do not include dipoles external to the brain, therefore any signal produced in the auditory periphery could be misattributed to the brain. Using the scalp surfaces created by Freesurfer, we remodelled the volume contained within the entire head using MNE in the first five subjects, and inspected the FFR>baseline difference map overlaid on the T1 anatomical map. We found no evidence of activity in the vicinity of the cochlea or auditory nerve.

### Asymmetry of the cortical FFR and behavioural relevance

We tested for rightward asymmetry by performing a two-tailed Wilcoxon signed-rank test on the difference between f0 amplitude during FFR in the right versus left ROI for each individual, using the data from the more accurate mixed model reported in Fig. 2. Rightward biases in MEG may be due to differences in signal cancellation due to underlying anatomy^47^; however, such artefacts would be unlikely to result in meaningful correlations with independently obtained behavioural or demographic variables, and if they were present, would likely affect a cortical ERF wave to a similar extent. We tested for rightward asymmetry as above in the time course of the left and right AC in the ERF-filtered data set; wave peaks were determined as the maximum within ±10 ms of the overall peak selected in the previous ERF analysis.

To test the hypothesis that right but not left AC f0 amplitude relates to training experience, we conducted one-tailed Spearman's correlation tests on the total instrumental and vocal training, and practice hours as self-reported in the MMHQ for subjects who indicated musical experience (*n*=11; left tail), and on f0 amplitude versus the age formal musical training started (left tail). We also evaluated correlations between f0 amplitude in each AC and fine pitch discrimination, and simple melody task scores, using all subjects (*n*=20).

## Additional information

**How to cite this article**: Coffey, E. B. J. *et al*. Cortical contributions to the auditory frequency-following response revealed by MEG. *Nat. Commun.* 7:11070 doi: 10.1038/ncomms11070 (2016).

## Supplementary Material

Supplementary Information Supplementary Figures 1-8

## Figures and Tables

**Table 1:** Explanatory power (root mean square levels of time courses of coefficient estimates) of the four pairs of ROIs over pairs of successive windows.

	0–12 ms, 12–24 ms		12–24 ms, 24–36 ms		24–36 ms, 36–48 ms		36–48 ms, 48–60 ms		60–72 ms, 12–84 ms	
	*Z*	*P*	*Z*	*P*	*Z*	*P*	*Z*	*P*	*Z*	*P*
CN	−3.34	<0.001*	−3.68	<0.001*	−1.139	0.13	−0.91	0.18	0.77	0.78
IC	−3.19	0.001*	−3.49	<0.001*	−1.699	0.05	−1.96	0.03	0.43	0.67
MGB	−3.38	<0.001*	−3.04	0.001*	−3.192	0.001*	−1.40	0.08	1.81	0.97
AC	−3.83	<0.001*	−3.57	<0.001*	−3.379	<0.001*	−2.59	0.005*	−1.70	0.05

AC, auditory cortex; CN, cochlear nucleus; IC, inferior colliculus; MGB, medial geniculate nucleus.

^*^Significant according to Wilcoxon-matched pairs test corrected for multiple comparisons within ROI, alpha=0.05/6).
