# Supplementary material for: Cortical contributions to the auditory frequency-following response revealed by MEG
Source: Nat Commun. 2016 Mar 24;7:11070. doi: 10.1038/ncomms11070 (PMC4820836; doi:10.1038/ncomms11070)
Supplement: Supplementary Information — Supplementary Figures 1-8 [file ncomms11070-s1.pdf]

Supplementary Figure 1

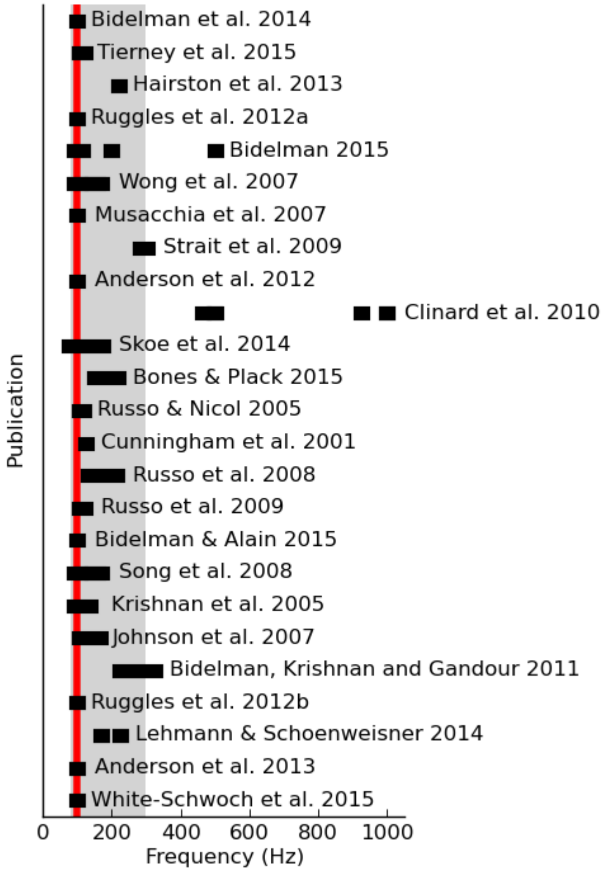

Supplementary Fig 1: The fundamental frequency of the stimulus used in this study (red line, 98Hz) as compared with those of stimuli used in relevant publications attributing FFR to brainstem structures (see Introduction). Static stimuli are centred on their f0, dynamic stimuli extend over their frequency range. The grey shaded area indicates the recommended f0 range to obtain a strong phase-locking response in ABR recording[1].

Supplementary Figure 2

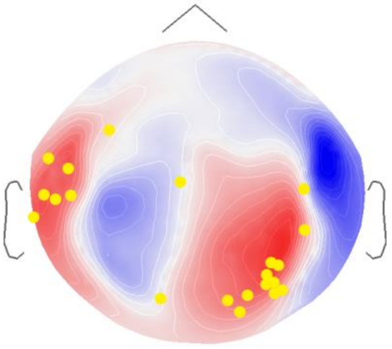

Supplementary Fig 2: Locations of channels of each subject used for single-channel MEG analysis (illustrated in Fig. 1 and Supplementary Fig. 3) are indicated with yellow circles. Channels were selected for maximum cross-correlation with the EEG signal within subject in order to best observe the MEG equivalent of the EEG onset response and FFR. Channel locations are plotted over the topography of a positive peak in the grand average fundamental frequency response, showing that most were in the right posterior or left anterior clusters where polarity was in phase with EEG. Channels selected for more than one subject are represented as slightly offset groups.

Supplementary Figure 3

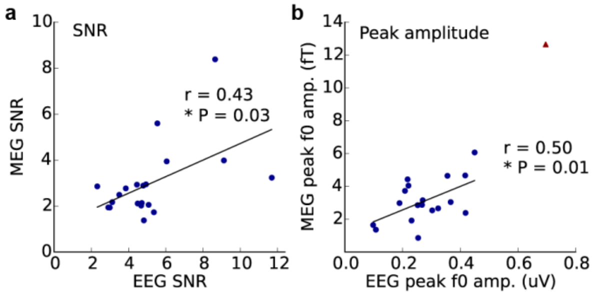

Supplementary Fig 3: Comparison of EEG and MEG single channel signal content demonstrates similarities and shared information despite differences in head shape and position that affect MEG. (**a**) The signal to noise ratio (i.e. FFR/baseline amplitude) of EEG and MEG signals was significantly correlated, as was (**b**) the peak amplitude of the fundamental frequency during the frequency following response. Linear trend lines are plotted for visualization purposes; one outlier (>3SD) was excluded in the calculation in (**b**) (red triangle)

Supplementary Figure 4

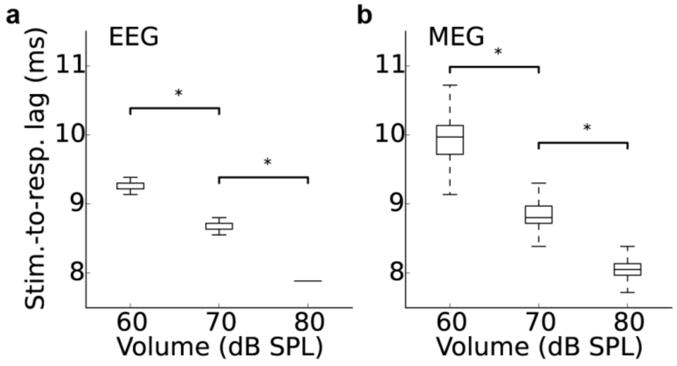

Supplementary Fig 4: Auditory stimulus amplitude manipulation to control for the possibility of signal contamination with a stimulation artifact[43] (n=1). Stimulus-to-response latencies are calculated using cross-correlation. (**a**) The EEG-ABR shows a previously reported decrease in stimulus-response latency with increasing stimulation volume (H(2)) = 27636, p <0.0001*). Planned Mann-Whitney U tests revealed that responses to 70dB sound were significantly slower than 80dB sound (U = 1.5e8*, r = 0.89), as were responses to 60dB when compared to those recorded in the 70dB condition (U = 1.5e8*, r = 0.91). (**b**) This phenomenon is also represented in the single-channel MEG data (H(2)) = 20494, p <0.0001*); 70dB responses were slower than 80dB (U = 1.4e8*, r = 0.76), and 60DB were slower than 70dB (U = 1.4e8*, r = 0.73). These results confirm the recorded MEG-ABR is of physiological origin.

Supplementary Figure 5

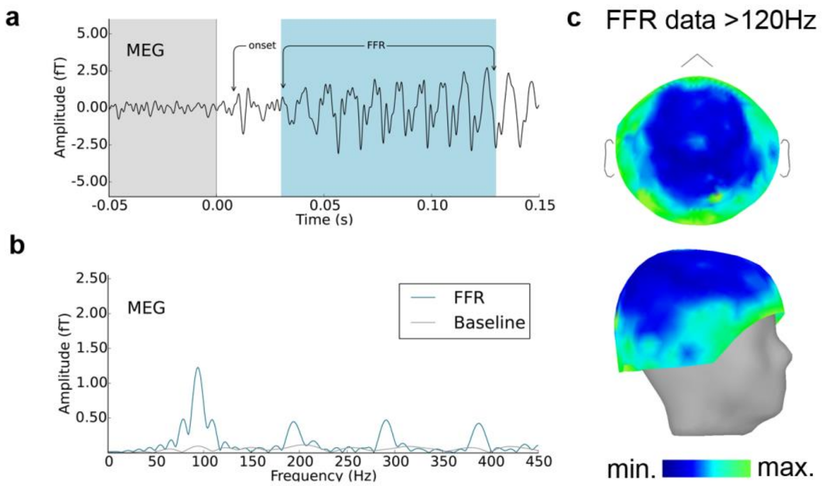

Supplementary Fig 5. Single-channel MEG-ABR grand averages when the MEG channel was selected by maximum correlation with 120Hz high-passed EEG channel data, in order to evaluate the presence of harmonic information in the MEG signal for future studies. a) Time domain and b) frequency domain representations show that the harmonics can be observed. c) Topographic distribution of energy between 120Hz and 450Hz (2nd to 4th harmonics of the 98Hz f0) suggests a predominantly subcortical origin.

Supplementary Figure 6

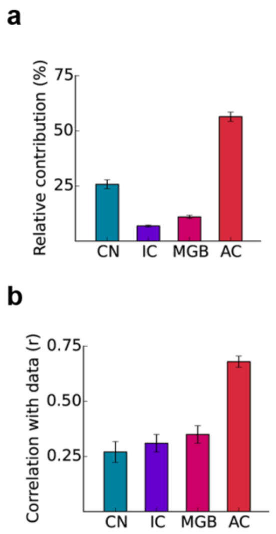

Supplementary Fig 6: (a) Relative percentage of explained signal in sensor space of each ROI projection, showing that the strongest contributor to the MEG-FFR is the AC, followed by the CN. (b) Correlations between each ROI simulation from **Fig. 4.a** and the topographic distribution in the recorded FFR data from **Fig. 4.b** for each subject; AC again shows the highest correlation. Error bars indicate standard error of the mean. Note that the relative weighting of sources in MEG does not imply identical weighting in EEG.

Supplementary Figure 7

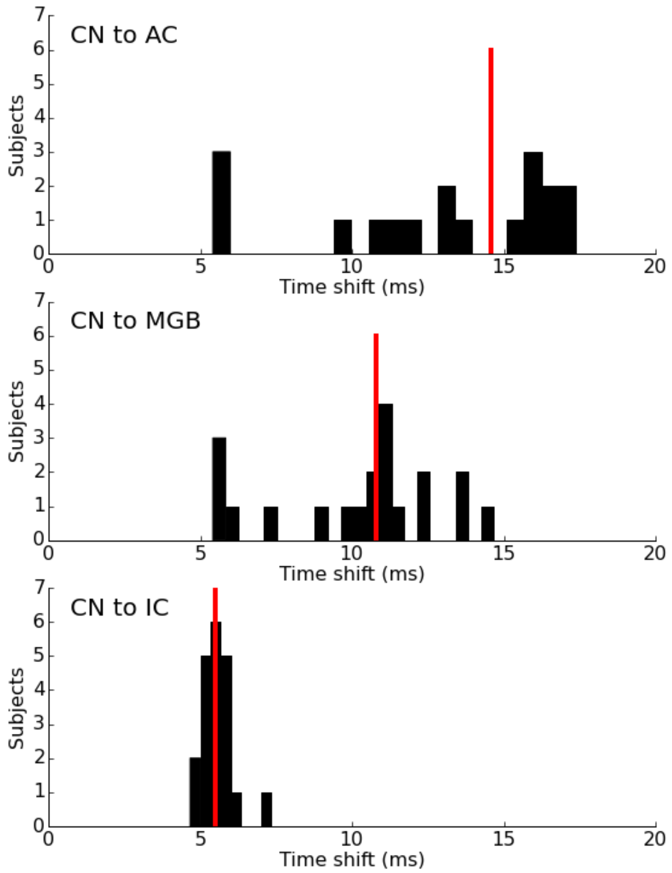

Supplementary Fig 7. Cumulative lag between ROIs, calculated by cross-correlation analysis of the time-varying coefficient estimates of successive ROIs (see also **Fig. 4**), each constrained to latencies less than the length of one period (0-9ms). **Bottom:** latency shift between CN and IC. **Middle:** cumulative latency shift between CN and IC, and IC and MGB. **Top:** cumulative latency shift between CN and IC, IC and MGB, and MGB and AC. Very few subjects show maximum correlations at or close to 0ms at each step, which would indicate poor separation of signals. These are presented for visualization primarily and not for statistical analysis.

Supplementary Figure 8

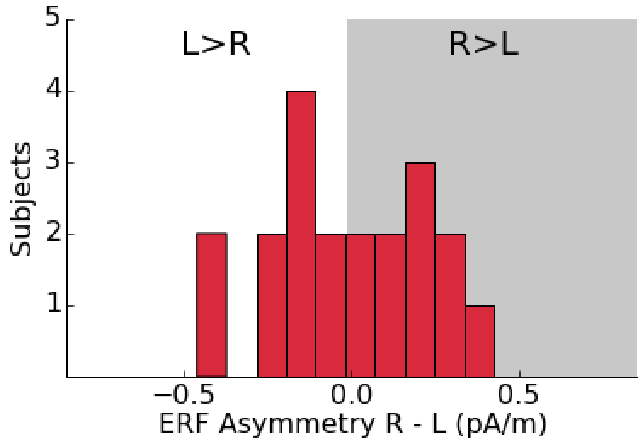

Supplementary Fig 8. Distribution of left-right amplitude differences in the first large ERF peak in the auditory cortex ROIs for each individual, using the mixed surface-volume MNE model (**Fig. 2**). Unlike its FFR counterpart (**Fig. 5b**), the ERF is not significantly asymmetric (Wilcoxon signed-rank test, Z = -0.04, P = 0.97).
